# Computer Vision Interaction Design in Sustainable Urban Development: A Case Study of Roof Garden Landscape Plants in Marine Cities

**DOI:** 10.3390/plants12183320

**Published:** 2023-09-20

**Authors:** Longlong Zhang, Chulsoo Kim

**Affiliations:** 1 Department of Marine Design Convergence Engineering, Pukyong National University, Busan 48513, Republic of Korea; 2 Department of Industrial Design, Pukyong National University, Busan 48513, Republic of Korea

**Keywords:** plants, green roof landscape, intelligent interaction design, computer visual design, marine cities

## Abstract

The rapid urbanization and the increasing need for sustainable development have led to the emergence of green roof landscapes in ocean cities. These rooftop gardens provide numerous environmental benefits and contribute to the overall well-being of urban dwellers. However, optimizing the design and interaction experience of green roof landscapes requires the integration of intelligent technologies. This paper explores the application of computer visual design techniques, specifically 3DMAX modeling and virtual reality, in the intelligent interaction design of green roof landscape plants in ocean cities. Designers can use computer visual design (3DMAX) and other technologies to interact intelligently with the roof landscape in order to improve landscape design. Through case studies, this indicated that computer vision is excellent for image processing of rooftop landscapes and also demonstrates a high degree of compatibility between computer vision and green rooftop landscape plant design in marine cities. This paper highlights the significance of intelligent interaction design and computer visual design techniques in optimizing the integration of green roof landscape plants in ocean cities. It emphasizes the potential of 3DMAX modeling and VR technology in creating immersive and engaging experiences for designers, users, and stakeholders alike. The findings contribute to the growing body of knowledge in the field of sustainable urban development and provide insights for designers, policymakers, and researchers seeking to enhance green roof landscapes in ocean cities. The dissertation highlights the potential of using computer vision design techniques to enhance the roof garden landscaping process and advocates for more efficient and effective ways to design, visualize, and improve rooftop gardens by utilizing software equipped with computer vision technology such as 3DMAX, ultimately contributing to the advancement of sustainable urban landscapes.

## 1. Introduction

Marine city roof landscape plants not only have excellent cooling and insulation effects but also can beautify the environment, purify the air, improve the local microclimate, and greatly increase the green coverage rate of the city [1]. They are a roof form worth vigorously promoting. With the progress of the times, the planting and design of rooftop landscape plants have become increasingly diverse, so studying rooftop landscape plants in marine cities is of great significance. To optimize the design and functionality of these green havens, the application of intelligent interaction design becomes crucial. This paper section aims to explore the concept of interaction design and its relevance in improving the user experience of rooftop gardens [2].

Interaction design is a multidisciplinary field that focuses on creating meaningful and engaging interactions between users and products, systems, or environments. In the context of green roof landscapes, interaction design involves the deliberate shaping of the user’s experience and interactions within the garden space. It encompasses various elements including spatial arrangement, plant selection, sensory stimuli, and user-centered considerations [3].

More and more people are studying green roof landscape plants in ocean cities. “Virtual Reality Applications in Landscape Architecture: A Review” by Elsayed et al. (2020) explored the use of virtual reality (VR) in landscape architecture, including green roof design and interaction [4]. It discussed the benefits and limitations of VR in creating immersive and interactive experiences and its potential in enhancing user engagement and understanding of green roof landscapes [5].

“Computer Vision-Based Techniques for Plant Phenotyping in Green Roofs” by Alsadik et al. (2020) focused on the application of computer vision techniques for plant phenotyping in green roofs [6]. It explored the use of image-processing algorithms and machine learning for plant growth analysis, including the measurement of leaf area, plant height, and biomass estimation. The findings provide insights into the potential applications of computer vision in optimizing plant growth in rooftop gardens. “Visualization and Virtual Reality Tools for Green Roof Design and Simulation” by Musa et al. (2018) explored the use of visualization and virtual reality tools in green roof design and simulation [7]. It discussed the integration of 3D modeling and VR technology to enhance the visualization and interaction of rooftop garden designs. The study emphasized the importance of user experience and engagement in the design process. Calheiros, C.S.C. believed that planting plants on green roofs has attracted considerable attention nowadays [8]. Subsequently, there is a hypothesis that native plants would be more adaptable to the environment, provide greater environmental benefits, and be more aesthetically pleasing than non-native plants. Although green rooftop landscape plants in ocean cities are widely planted today, there are still some aspects that need to be improved.

The research methodology of this paper involves a combination of literature review, case studies, and empirical analysis. The literature review provides a comprehensive understanding of the existing knowledge and research in the field of intelligent interaction design and computer visual design techniques [9]. Case studies and examples are used to illustrate the practical application of these techniques in the context of green roof landscape plants in ocean cities. Additionally, empirical analysis considers the gathered data and evaluates the effectiveness of the implemented computer visual design techniques through user evaluations, feedback, and quantitative measures. The research methodology ensures a comprehensive exploration of the research objective and provides valuable insights into the integration of computer visual design techniques in green roof landscape plants [10].

By employing intelligent interaction design principles, rooftop gardens can offer more than just a visual spectacle. They can become immersive and interactive spaces that stimulate curiosity, learning, and a deeper connection with nature. Intelligent interaction design takes into account the diverse needs and preferences of users, fostering engagement, and promoting a sense of well-being [11]. For instance, the arrangement of plants within the garden can be strategically designed to create a sensory journey, engaging users through a variety of colors, textures, and fragrances. The pathways and seating areas can be optimized to provide opportunities for exploration, relaxation, and social interaction. By leveraging intelligent interaction design, rooftop gardens can cater to the diverse needs of users whether they seek a peaceful retreat, a space for social gatherings, or an educational experience [12].

Furthermore, interaction design can be enhanced through the integration of computer visual design techniques such as 3D modeling and virtual reality. These technologies enable designers and users to visualize and interact with green roof landscapes in a dynamic and immersive manner. Three-dimensional modeling allows for realistic representations of the garden space, facilitating the evaluation of plant placements, spatial arrangements, and overall design concepts. VR technology, on the other hand, offers users the opportunity to virtually explore and interact with the rooftop garden, offering a deeper understanding of the plant ecosystem and experience of the space from different perspectives [13].

Computer vision is also a commonly used technology nowadays, and its use regarding green rooftop landscape plants in ocean cities is also a trend. Computer vision has excellent image-processing functions, which can identify the distribution of various samples in the landscape. This is very helpful for the design of rooftop landscape plants in ocean cities. In this experiment, computer vision technology was also used to identify the greening rate of green rooftop landscape plants in ocean cities. From the results, it can be found that computer vision is still very effective in analyzing the greening of green landscape plants in marine cities [14].

The use of new tools and design methods utilizing computer methods in green roof planting landscape design helps designers to create more precise and knowledge-based greening designs. This article describes the application of computer vision design techniques, especially in the field of roof garden landscaping, using software such as 3DMax2020. This involves using the capabilities of such software to digitally model and visualize roof garden layouts, plant arrangements, and other landscape elements. These tools, often rooted in computer vision principles, empower designers and architects to meticulously simulate, model, and visualize every intricate facet of landscapes, structures, and objects. By incorporating these techniques into green roof landscaping, we aim to facilitate a more precise, informed, and knowledge-driven approach to greening design.

## 2. Intelligent Interaction of Green Roof Landscape Plants in Ocean Cities Based on Computer Vision

### 2.1. Overview of Green Roof Landscape Plants in Ocean Cities

Just like other architectural landscapes and forests, the green roof landscape of ocean cities is a combination of water, mountains, and rocks based on terrain and natural features to form a garden. In urban greening, roof greening accounts for a large proportion and is also known as a plant that leaves the soil environment. With the increase of buildings and population, people’s desire for natural greening has become more urgent [15]. Instituting greenery on roofs is thriving in a unique form. Not only is it more closely integrated with architectural plants, but it also enriches the aesthetic of the building, providing people with a wider view and more green space with landscapes. Roof greening is carried out within a limited space and is subject to many limitations. Therefore, detailed planning and design are required for the elements, structure, volume, and style of roof greening [16]. 

In addition to designing according to the technical principles of architectural design, roof greening in ocean cities also needs to consider the structure of the roof, the waterproof and drainage structure of the roof, the growth characteristics of plants, and the growth techniques of plants. These are all technical requirements for creating roof landscapes. The key to the success of roof greening construction lies in the bearing capacity of the roof, the ability of the roof for drainage and waterproofing, and the combination of greening plant species [17]. In order to solve the problem of bearing capacity in roof greening, landscape plants with too large a volume should not be installed in the roof landscape. In the treatment of the site, it should be mainly flat, and the roof landscape can be designed according to the natural conditions of the roof to meet the greening needs of the building landscape and increase the level of landscape space. 

### 2.2. Design Principles for Rooftop Landscape Plants in Marine Cities

Natural layout: Most of the plants in the roof landscape are arranged in a natural way so that the roof landscape can form a natural garden effect. The natural layout of the roof landscape is meant to match the roof landscape with the surrounding buildings and water bodies and then combine trees and flowers to form a dense natural landscape [18]. 

Rule-based layout: In rule-based layout, the designer needs to pay attention to the decoration of plants so that the plants in the garden form a regular and hierarchical plant configuration. This plant configuration has a fresh and solemn landscape atmosphere, which can give people a good feeling. 

Mixed layout: The mixed roof landscape not only possesses the landscape characteristics of natural and regular layout but also has its own unique style. In the changes of spatial composition points and surfaces, it forms a variety of spatial levels. This type of rooftop landscape is designed to enhance the continuity of the plant landscape and emphasize the diversity of landscape plants, thereby closely combining different types, styles, and landscapes to form an extremely beautiful rooftop landscape [19]. 

**Figure 1:**
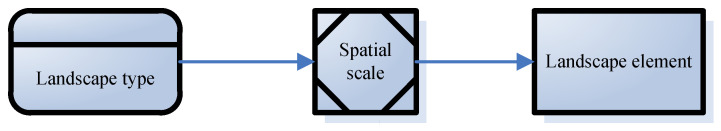
The composition of green roof landscape in an ocean city.

The composition of the green roof landscape in marine cities is shown in Figure 1. 

### 2.3. Role of Rooftop Landscape Plants

Enhancing urban climates in ocean cities: Rooftop landscape plants have demonstrated their capacity to contribute significantly to urban climate improvements. These plants play a pivotal role in absorbing particulate dust and mitigating the presence of toxic gases in the atmosphere, simultaneously regulating the humidity levels within urban environments. Moreover, they serve as effective buffers for reducing noise pollution in residential zones, showcasing a notable noise-reduction efficacy. Through the process of photosynthesis, green plants exhibit an impressive ability to sequester substantial quantities of carbon dioxide from the surrounding atmosphere, thereby acting as a countermeasure against the formation of urban “heat islands”. Additionally, these rooftop plants offer a protective function, preserving the integrity of the waterproofing layer on rooftops, consequently extending the operational lifespan of the roofs [20].

Beautifying the urban air landscape: The development of ocean urbanization is very rapid. If different forms of rooftop landscape plants can be arranged at the top of these buildings, then above the city, there would not be just a lifeless concrete roof. Relevant data show that if there is green comprises 25% of the sight line, it can make people feel happy. The existence of rooftop landscape plants can precisely meet this demand, which can regulate people’s psychological state and effectively improve the mental outlook of urban populations [21]. The multifaceted roles of rooftop landscape plants extend beyond mere visual enhancement. These botanical elements, carefully selected and positioned, act as urban ecotones, seamlessly blending nature into the urban fabric. Beyond their immediate visual appeal, these plants play an active role in fostering biodiversity by providing habitats for various insects and birds that contribute to a balanced ecosystem. Furthermore, they serve as natural air purifiers, absorbing pollutants and releasing oxygen, thereby improving the overall air quality of the city. This harmonious coexistence of greenery and urbanity not only delights the senses but also creates a harmonizing effect that resonates with the aspirations of modern urban living.

Having a good insulation effect: Due to direct sunlight in summer, roofs without greenery have much higher temperatures than the outside. Moreover, due to the color and material of the roofs, the heating rate varies, with some roofs reaching a heating rate of over 80%. However, on a roof with green vegetation, the surface temperature of the roof can be effectively reduced due to the blocking of heat by leaves and the emission of heat by transpiration. Therefore, the indoor temperature of the building can be effectively controlled in the hot summer [22]. If the roof is arranged with a carpet-like lawn and with a certain number of crawling plants placed on the walls, it can effectively reduce indoor temperature and mitigate a lot of electricity consumption. Plants’ ability to act as natural coolants during sweltering summers is facilitated through their shading effect, which reduces solar heat gain on rooftops. This not only directly contributes to lower indoor temperatures but also translates into reduced energy consumption for air conditioning. Moreover, these plants play a pivotal role in stormwater management by absorbing rainwater, thereby reducing runoff and the strain on drainage systems. In stark contrast, in winter, this covered roof can provide excellent insulation. The dynamic role of rooftop landscape plants as thermal regulators underscores their significance in moderating indoor temperatures. By forming a protective layer against heat loss, they contribute to energy savings by reducing the need for excessive heating. This dual-season insulation effect aligns with the ethos of sustainability and resource optimization, further reinforcing the instrumental value of rooftop landscape plants in contemporary urban design.

### 2.4. Computer Visual Design Techniques

Computer visual design techniques encompass various methods and tools used to create and manipulate visual elements in digital environments. Two key technologies in this realm are 3D modeling and virtual reality (VR). Three-dimensional modeling involves the creation of three-dimensional digital representations of objects or spaces, allowing for a realistic and interactive visualization. VR, on the other hand, immerses users (or the design team responsible for creating and implementing the rooftop garden design) in a simulated environment, enabling them to interact with and experience a virtual world [23].

**Figure 2:**
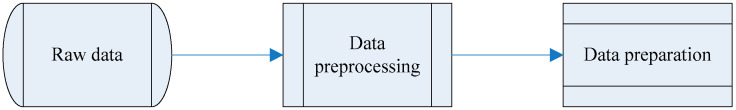
Computer vision system’s data-preparation phase.

In computer vision, image processing refers to the analysis and manipulation of digital images using algorithms and computational methods. It involves tasks such as image enhancement, segmentation, feature extraction, and object recognition. By extracting meaningful information from images, computer vision techniques enable the understanding and interpretation of visual data. The data-preparation stage of the computer vision system is shown in Figure 2.

**Figure 3:**
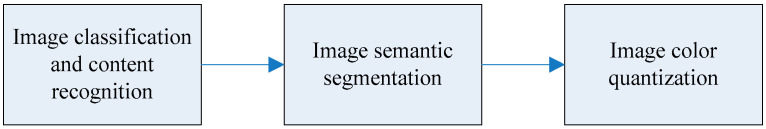
Methods of computer vision image processing.

The method of computer vision image processing is shown in Figure 3.

Computer visual design techniques refer to the utilization of advanced software tools, often based on computer vision technology, to create and manipulate visual elements in a digital environment. These techniques enable designers and architects to simulate, model, and visualize various aspects of landscapes, structures, and objects in a highly detailed and interactive manner.

In this thesis, highlighting the application of computer visual design techniques, specifically using software like 3DMax, in the field of rooftop garden landscaping. This involves using the capabilities of such software to digitally model and visualize rooftop garden layouts, plant arrangements, and other landscape elements. The software’s computer vision capabilities can assist in various ways:

Three-dimensional modeling: Computer visual design software like 3DMax allows designers to create detailed three-dimensional models of rooftop landscapes. This modeling can encompass everything from the layout of plants and structures to the overall spatial arrangement, providing an accurate representation of how the final garden would look.

Realistic visualization: These software tools enable the creation of high-fidelity visualizations that closely resemble real-world conditions. Designers can incorporate realistic textures, lighting, and environmental factors, giving stakeholders a clear and vivid understanding of the proposed rooftop garden’s appearance.

Simulation and analysis: Computer visual design software often includes simulation features that can be used to analyze different scenarios. For rooftop garden landscaping, this could involve simulating the growth of plants over time, assessing factors like sunlight exposure, wind patterns, and how the garden might evolve seasonally.

Iterative design: The digital nature of these techniques allows for quick and easy modifications to the design. Designers can experiment with various plant placements, materials, and layout configurations, rapidly refining their ideas to achieve the desired outcome.

Stakeholder communication: These visualizations can serve as effective communication tools between designers, clients, and other stakeholders. By providing a realistic preview of the proposed rooftop garden, these techniques facilitate better understanding and decision making.

In essence, this thesis underscores the potential of utilizing computer visual design techniques to enhance the process of rooftop garden landscaping. By harnessing software equipped with computer vision technology, such as 3ds Max, designers can advocate for a more efficient and effective approach to designing, visualizing, and refining rooftop gardens, ultimately contributing to the advancement of sustainable urban landscapes.

### 2.5. The Landscape Design Process for Roof Gardens

The landscape design process for roof gardens involves a series of systematic steps aimed at conceptualizing, planning, and creating green spaces on the rooftops of buildings. This paper emphasizes the utilization of computer visual design techniques, particularly software with computer vision technology like 3ds Max, to enhance and facilitate this process. The following is an overview of the typical steps in the landscape design process for roof gardens:

Site analysis and assessment: The process begins with a comprehensive evaluation of the rooftop’s characteristics. This includes assessing factors such as sunlight exposure, wind patterns, structural integrity, load-bearing capacity, and existing utilities. Computer visual design techniques can aid in creating accurate digital models of the rooftop, allowing designers to analyze these factors in detail.

Conceptualization and ideation: Designers brainstorm and develop initial ideas for the rooftop garden. This phase involves considering the client’s preferences, functional requirements, and aesthetic goals. Computer visual design software enables the creation of digital sketches and preliminary visualizations that help translate conceptual ideas into tangible representations.

Design development: The chosen concepts are refined and developed further. This involves specifying plant selections, hardscape elements (such as pathways and seating areas), and other design elements. Computer visual design techniques allow for the creation of detailed 3D models that provide a realistic preview of the garden’s layout, helping designers and clients visualize the final outcome.

Visualization and simulation: This step involves using computer visual design tools to create high-quality visualizations and simulations. Designers can apply realistic textures, lighting, and environmental effects to the digital models. Additionally, simulations can be used to predict how plants will grow over time, how shadows will change during different times of the day, and how the garden will evolve with the changing seasons.

Refinement and iteration: Computer visual design software allows for easy modifications to the design. Designers can experiment with various plant arrangements, colors, materials, and spatial configurations. Iterative refinement ensures that the design aligns with the client’s vision and functional requirements.

Documentation and communication: Detailed plans, sections, and elevations are generated using the software tools. These documents serve as crucial references for construction and installation. Additionally, the high-quality visualizations facilitate effective communication with clients, stakeholders, and contractors, ensuring a shared understanding of the design intent.

Implementation and maintenance: Once the design is finalized, the actual construction of the rooftop garden takes place. Proper installation of plants and hardscape elements follows the detailed plans and specifications generated during the design phase. Ongoing maintenance ensures the garden thrives and continues to contribute positively to the urban environment.

### 2.6. Benefits and Applications in Interaction Design

The application of computer visual design techniques, specifically 3D modeling and VR, offers numerous benefits and applications in the field of interaction design, particularly in rooftop gardens [24].

Enhanced visualization: 3D modeling enables designers and users to visualize rooftop gardens in a realistic and immersive manner. It allows for a better understanding of spatial arrangements, plant placements, and design concepts. Users can explore the garden from different perspectives, fostering a deeper connection with the space and facilitating informed design decisions [25].

Interactive exploration: VR technology provides an unparalleled opportunity for users to interact with rooftop gardens. By simulating the experience of being in the garden, users can virtually walk through the space, interact with plants, and experience various design elements. This interactive exploration promotes engagement and enables users to envision the potential of the rooftop garden [26].

Iterative design process: Computer visual design techniques facilitate a more iterative and collaborative design process. Designers can create, modify, and refine 3D models of the rooftop garden, allowing for quick prototyping and testing of different design iterations. Users can provide feedback and actively participate in the design process, resulting in more user-centered and optimized designs.

Educational and informative experiences: Computer visual design techniques can be leveraged to create educational and informative experiences in rooftop gardens. Through 3D modeling and VR, users can learn about different plant species, their characteristics, and their ecological relationships [27]. This interactive learning experience fosters environmental awareness and promotes sustainable practices.

User engagement and well-being: The immersive and engaging nature of computer visual design techniques enhances user experiences in rooftop gardens. By incorporating interactive elements such as touch-based interactions or gamified activities, users can actively engage with the environment, fostering a sense of well-being, curiosity, and connection with nature.

Computer visual design techniques, including 3D modeling and VR technologies, offer numerous benefits and applications in the field of interaction design for rooftop gardens. They enable enhanced visualization, interactive exploration, iterative design processes, educational experiences, and increased user engagement. By leveraging these technologies, rooftop gardens can provide immersive and engaging experiences that promote sustainable design and improve the overall well-being of users [28].

## 3. A Case Study of the Application of Computer Visual Design to Green Rooftop Landscapes in Marine Cities

**Figure 4:**
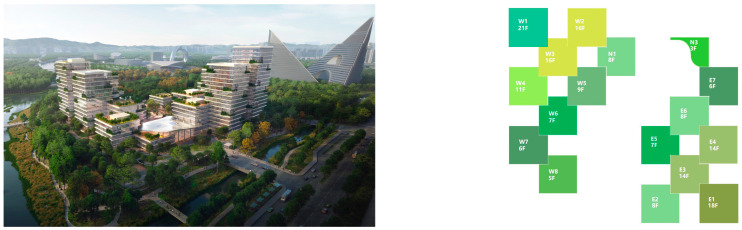
Location Map of Alibaba Corporate Headquarters Building and Roof Garden.

The Alibaba office building headquarters is a landmark building located in Hangzhou, China, and is home to the headquarters of Internet giant Alibaba. The building was designed by the internationally recognized architectural firm, Foster + Partners, and is an office and a commercial, cultural, and recreational complex. There are 15 roof gardens in the overall park, with a minimum area of 390 square meters and a maximum area of 878 square meters (As in Figure 4).

This paper takes the rooftop garden of Alibaba’s headquarters building as a case study to conduct in-depth research on the application of computer visual design techniques, specifically computer vision and 3D rendering, in the intelligent interaction design of green roof landscape plants in ocean cities. The aim is to explore the potential of these technologies in optimizing the integration of green roof landscape plants and improving the overall landscape design.

To begin, computer vision techniques were employed to analyze and process images of the rooftop garden. By capturing high-resolution images and utilizing image-processing algorithms, various aspects of the rooftop garden, such as plant composition, spatial arrangement, and overall greening rate, were measured and evaluated. Computer vision enabled efficient and accurate data collection, providing valuable insights into the effectiveness of green roof landscapes in marine cities. Next, 3D rendering techniques were utilized to create detailed and realistic representations of the rooftop garden. By translating design drawings and plant simulations into three-dimensional models, designers are able to visualize and analyze the interactive design aspects of the plant landscapes. Herein, this included evaluating the placement of different plant species, assessing their visual impact, and understanding the ecological relationships within the rooftop garden.

The research focuses on several key aspects. Firstly, the greening rate of plants in the rooftop garden was measured and analyzed using computer vision techniques. By quantifying the coverage and distribution of plants, the effectiveness of the green roof landscape in providing environmental benefits, such as heat insulation and carbon sequestration, was able to be assessed. Furthermore, the composition of the rooftop garden’s plant species was examined. Computer vision techniques aided in identifying and categorizing the various plants present in the rooftop garden. This analysis provided valuable information on the biodiversity and ecological balance of the green roof landscape. It also enables designers to make informed decisions regarding plant selection, considering factors such as adaptability to marine climates, resistance to saltwater exposure, and compatibility with the overall design concept. The interactive design of the plant landscapes was explored based on the 3D rendering models. By virtually experiencing the rooftop garden through VR technology, designers, users, and stakeholders can engage in interactive exploration and provide feedback on the design. This user-centered approach fosters participatory design, allowing for adjustments and improvements to be made based on user preferences and needs.

### 3.1. Simulation Experiment of Green Roof Landscape Plants in Marine Cities under Computer Vision

Ocean city data and computer technology have become important sources and methods for measuring landscape quality [29]. With the continuous progress and application of computer vision algorithms, landscape preference research has now developed towards large-scale, quantitative, and high-precision directions. The use of computer vision algorithms to measure landscape spatial quality and public perception preferences has been explored in multiple fields and scales, and various attempts have been made in the application of different algorithms [30]. At present, there is relatively little research in this field, and the research direction is mostly focused on the measurement of the green vision rate. There are also some shortcomings in the application and innovation of algorithms. Therefore, it is necessary to study a multi-dimensional landscape image quantification measurement method based on public perception and to use various computer vision algorithms to parameterize green roof landscape plant images. By extracting various landscape features from images and summarizing the dimensional structure of landscape images from multiple perspectives, the public’s perception preferences can be analyzed. 

Machine learning is often used in computer vision algorithms. Machine learning utilizes the set Q as training data. The best assumption in the assumed space is h, and N(h) is used to represent the initial probability of hypothesis h before the untrained dataset Q, while P(Q) reflects the probability of hypothesis h holding after setting the training data Q. The calculation method for probability is shown in Formula (1):

(1) $$P(Q)=\frac{N(h)}{h}$$

In the process of machine learning, the assumption of maximum likelihood is called the maximum posterior hypothesis, and the calculation method is shown in Formula (2):

(2) h=arg min P(Q)

By combining Formulas (1)–(3), the following can be obtained:

(3) $$h=arg\;min\frac{N(h)}{h}$$

Computer vision techniques offer a powerful means to extract valuable landscape features through the utilization of image segmentation algorithms. One particularly useful metric is the green view index (GVI), which provides a quantitative measure of the greening level in ocean cities’ landscapes. By quantifying the panoramic green view rate, it is possible to assess the aesthetic value of these cities and understand the significant impact of greening rates on people’s overall life satisfaction and well-being [31]. And the study of greening rates also contributes to the refinement and development of interactive landscaping [32].

**Figure 5:**
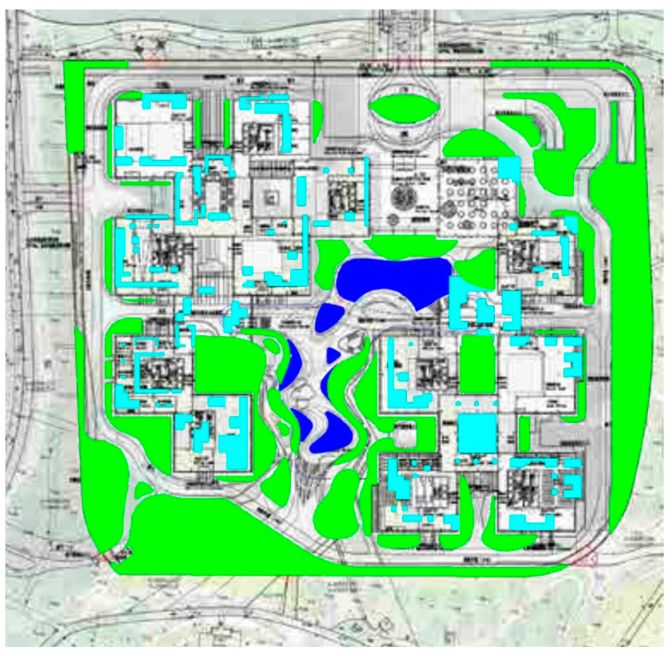
Roof garden green space map.

**Figure 6:**
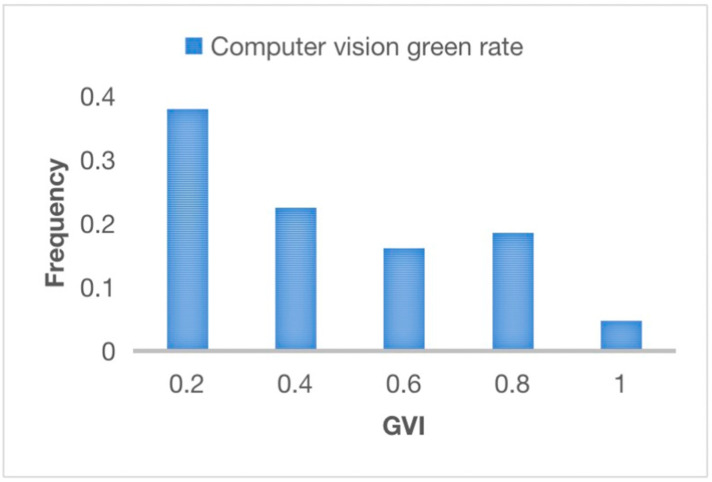
Distribution of green apparent rate of samples of green roof landscape plants in the rooftop garden of the Alibaba corporate headquarters building.

Figure 5 and Figure 6 provides a visual representation of the numerical distribution of the green visibility rate across all samples of green roof landscape plants in marine cities. This data visualization offers valuable insights into the effectiveness of these rooftop gardens in increasing the overall greening rate, further highlighting the significance of intelligent interaction design and computer visual design techniques in optimizing the integration of green roof landscape plants in ocean cities. By utilizing computer vision algorithms to extract landscape features and quantify the greening rate through the green view index, this research highlights the vital role of intelligent interaction design and computer visual design techniques in optimizing the integration of green roof landscape plants in ocean cities.

The analysis of the data presented in Figure 6 provided insightful observations regarding the distribution of green landscape plant samples based on their greening rates. It is evident that the sample group with a greening rate of 0.2 represents the largest proportion, accounting for a significant 38% of the total samples. Additionally, the green landscape plant samples with a greening rate of 0.2 make up 22.5% of the overall samples. In contrast, the differences in proportions among the samples with greening rates ranging from 0.6 to 0.8 are not statistically significant. Remarkably, when the greening rate reaches 1, the sample proportion is the smallest, comprising only 4.8% of the total.

These experimental findings shed light on the greening rates of green roof landscape plants in the Alibaba corporate headquarters building roof garden, indicating a moderate level of greening. The results highlight the effectiveness of computer vision in observing and evaluating the greening levels of ocean city rooftop landscapes through advanced image-processing techniques and other technologies. This reflects the success of intelligent interaction enabled by computer vision, which played a significant role in obtaining relatively accurate greening rate measurements.

The experiment’s outcomes further emphasize the significance of intelligent interaction design and computer visual design techniques in optimizing the integration of green roof landscape plants in ocean cities. By leveraging computer vision, designers can interact intelligently with the rooftop landscape, gaining precise measurements of greening rates. This intelligent interaction allows designers, users, and stakeholders to make informed decisions regarding landscape design, fostering the creation of aesthetically pleasing and environmentally beneficial green roof environments.

### 3.2. Analysis of Plant Landscape Interaction Design for Rooftop Garden of Alibaba Corporate Headquarters Building

#### 3.2.1. Overall Analysis

**Figure 7:**
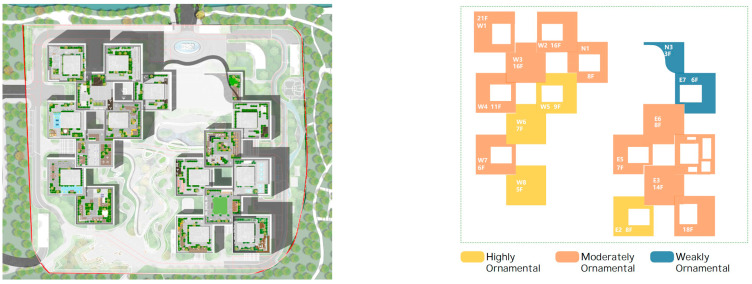
Three-dimensional floor plan and ornamental analysis diagrams.

The analysis of the roof gardens’ ornamental intensity reveals that among the studied samples, there are four roof gardens categorized as having strong ornamental qualities, nine classified as medium, and two classified as weak (As shown in Figure 7). The areas with strong ornamental roof gardens exhibit a relatively high level of greening, aligning with the greening rate analysis discussed in the previous section.

The roof gardens encompass diverse spatial types, including multifunctional composite integrated spaces, public open spaces, semi-public open spaces, and private spaces. By distinguishing the characteristics of these roof gardens, they can be primarily categorized into four functional modules: negotiation spaces, observation decks, multi-person party areas, and VR virtual experiences. The careful arrangement and pairing of each functional module contribute to enhancing the interaction between individuals and the green roof landscape plants.

The negotiation space serves as a gathering area where people can engage in discussions and collaborative activities while being surrounded by the soothing presence of plants. The observation deck provides an elevated vantage point for individuals to appreciate the panoramic view of the green roof landscape, fostering a deeper connection with nature. The multi-person party area offers a social space where people can come together, socialize, and enjoy the vibrant ambiance created by the greenery. Finally, the inclusion of VR virtual experiences allows users to immerse themselves in virtual simulations of the rooftop gardens, providing an interactive and engaging platform to explore and interact with the plant landscapes.

#### 3.2.2. Interactive Plantscape Design

Hangzhou, situated in the subtropical monsoon zone, experiences distinct seasons, abundant rainfall, and a wide variety of plant species suitable for growth. The greening efforts in Hangzhou follow a people-oriented approach, emphasizing ecological diversity, scientific principles, and artistic aesthetics. The selection of landscape tree species with ornamental value aims to meet functional needs such as accessibility, line-of-sight permeability, boundary richness, and interactivity. The design of rooftop gardens focuses on simplicity, aligning with the natural and functional requirements of green spaces. Attention is given to the interdependence between plants, ensuring a harmonious and visually appealing plant composition that incorporates color coordination, shapes, textures, and sounds to create a captivating landscape effect. The open entrance space of the rooftop garden employs ecological treatments to create a sense of grandeur. The tree species chosen should meet the requirements of the roof garden in terms of load bearing and maintenance management. Shaded activity areas are created through large trees with high points of support. The shrub groundcover is carefully trimmed to follow a natural curvature, which helps to soften the undulating and staggered heights, thereby mitigating the stark lines of the building. Comfortable lawns and natural flower paths are utilized to establish a serene leisure space and a tranquil resting area. Along the street, the display surface prominently features tall, straight, and large trees that serve as structural elements for the entire space. Combined with the building’s visual interface, this creates a cohesive and expressive landscape, leaving a powerful visual impression on observers. Through the curved pruning of shrubs and ground cover, the building boundary is softened, and there is no lack of rich changes in the sense of sequence, which highlights the nobility and sophistication.

There are also shrubs, ground covers, and trees, as shown in Table 1.

**Figure 8:**
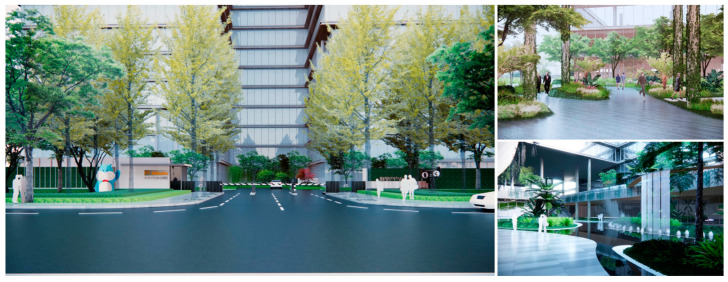
Three-dimensional rendering of roof garden entrance.

**Figure 9:**
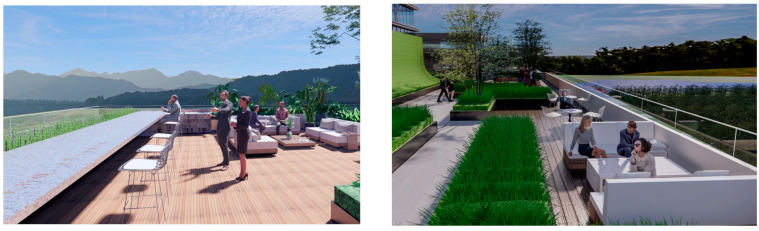
Rendering of Rooftop Observation Deck.

The open activity space adopts a planting pattern that combines comfortable sparse forests and lawns, resulting in a leisurely landscape along the pathways. This design not only prioritizes walking comfort but also encourages people to engage with nature and participate in outdoor activities. The selection of large-crowned and shade-providing camphor trees creates pleasant, shaded areas where visitors can seek shelter from rain and engage in communication activities. The semi-open waterfront space accommodates the growth of lotus and cattails, creating a beautiful and natural water landscape. Along the shore, a wide variety of aquatic plants are planted to soften the water’s edge and purify the water body. Additionally, the strategic placement of reeds around the artificial wetland allows visitors to have an up-close view of the garden without obstructing their line of sight, enhancing visual interaction (as shown in Figure 8 and Figure 9).

**Figure 10:**
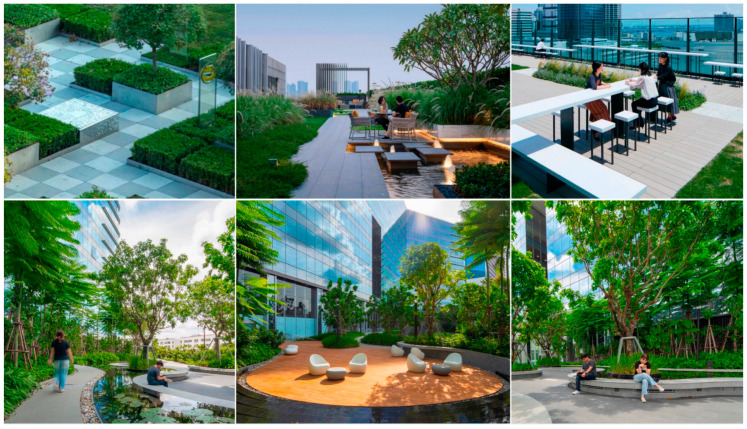
Roof garden 3D effect.

The semi-open space features sparse forests dominated by green plants such as zelia and calamus, allowing visitors to experience the ever-changing charm of natural plant growth along their walking path and providing a closer connection to nature. The addition of other native tree species enriches the diversity of the landscape, creating a suitable habitat for birds to survive and forage. This in turn attracts birds and offers visitors a pleasant visual interaction experience, accompanied by the sounds of bird calls for an enhanced auditory experience. In the semi-enclosed space, plants like yew, magnolia, and camphor are planted densely, forming a hidden landscape that tantalizes visitors’ curiosity (as shown in Figure 10).

**Figure 11:**
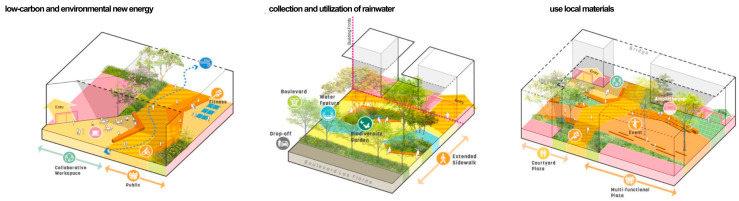
Schematic diagram of the ecological diversity of rooftop gardens.

The rooftop garden of Alibaba’s headquarters building uses solar energy, wind energy, and other low-carbon and environmentally friendly new energy to increase the utilization rate and utilizes permeable paving, green roofing, soil and water conservation plants, etc. In terms of the selection of materials for the structures, paving, and facilities, Bengoshi stone, wood, and other materials are used to create a comfortable and pleasant environment and effectively protect the ecological environment. In the selection of materials for structures, paving, facilities, and sketches, the Bengoshi materials were selected to create a pleasant environment and effectively protect the ecological environment and to comprehensively utilize rainwater resources, conserve water, and improve the ecological environment with the use of innovative technology and techniques that are in line with the sustainability of the ecological environment and with local characteristics that are more harmonious and natural (as shown in Figure 11).

**Figure 12:**
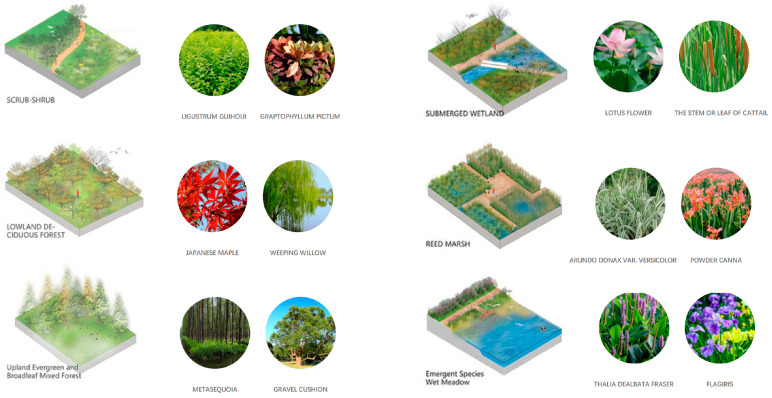
Roof garden plant analysis chart.

The overall design of the rooftop garden emphasizes interactive plant landscapes, allowing people to experience the dynamic changes and charm of plant growth during their restful walks or conversations throughout different seasons. The characteristics of the plant landscape are harnessed to stimulate visual, tactile, auditory, and behavioral interactions, igniting human imagination and increasing visitors’ interest in getting closer to nature. This design approach fosters a stronger connection between humans and nature, creating a more intimate relationship. Figure 12 provides a plant analysis diagram to further illustrate the diverse plant composition and layout within the rooftop garden.

#### 3.2.3. Interactive Landscape Lighting Design

**Figure 13:**
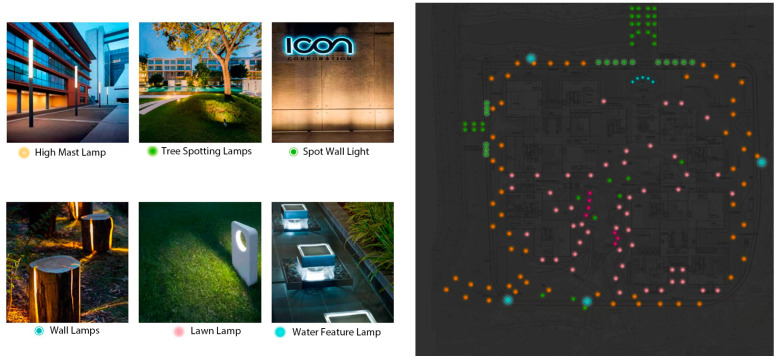
Rooftop garden plant landscape lighting design analysis diagram.

In the context of the rooftop garden at Alibaba’s corporate headquarters building, the plant landscape lighting design plays a crucial role in enhancing its interactivity with people, especially during nighttime hours. The lighting design aims to highlight the unique characteristics of the plant landscape while creating a captivating environmental atmosphere. Various factors such as plant height, overall texture, branches, leaves, and the positioning of lamps and light sources are considered in the design process. By employing suitable lighting methods, the desired visual effects were achieved, transforming the rooftop garden into an artistic and decorative space. The roof garden plant landscape lighting design analysis diagram is shown in Figure 13.

The landscape lighting at Alibaba’s headquarters utilizes techniques such as light superposition, flow, translucency, and refraction to craft a highly imaginative sensory experience. Lamps are strategically concealed using techniques such as plant shading, light trough hiding, and low-level covering, creating an enchanting play of light that connects the area with nature. Suspended or silhouetted lighting effects further add to the magical ambiance of the rooftop garden.

A critical aspect of the plant lighting design is the choice of light source color. Different light sources can elicit varying color appearances and details in plants, evoking distinct emotions and sensations. The design team carefully selected colors to harmonize with each plant’s inherent color and form, taking into account the unique structure of each tree. For spreading branches, up-lighting is used to illuminate the trunk and branches effectively, creating stunning effects for species like camphor and magnolia. In contrast, coniferous species with dense crowns near the ground require spotlights to illuminate their canopies from the periphery, showcasing seasonal changes throughout the year.

The plant landscape in the rooftop garden undergoes transformations in appearance with varying weather, seasons, and time of day. This dynamism was thoughtfully embraced in the lighting design, with corresponding adjustments made to highlight specific elements of the garden based on the season. During spring, summer, and fall, the focus may be on showcasing the lush crowns and foliage, while in winter, the emphasis shifts to the striking branches and tree trunks. This approach enhances the interactivity between visitors and the ever-changing plant landscape, allowing people to immerse themselves in the garden’s unique atmosphere and the visual spectacle presented by different seasons.

To achieve a human-centric design, the lighting plan takes into account human physiological and psychological characteristics. The composition of light in the landscape design was carefully considered to evoke emotions and interactions, making visitors feel more connected to the plants and the environment. By thoughtfully combining technological prowess with a deep understanding of human needs, the rooftop garden at Alibaba’s corporate headquarters exemplifies the integration of intelligent interaction design and computer visual design techniques, resulting in an engaging and visually captivating green roof landscape in the context of an ocean city. The lighting design not only enhances the user experience but also contributes to the overall well-being of urban dwellers, providing insights for future green roof landscape projects in ocean cities seeking to incorporate intelligent technologies for sustainable urban development.

### 3.3. Chapter Summary

Through computer vision image-processing techniques, the greening rate of the rooftop garden at Alibaba’s headquarters building was accurately determined. These findings demonstrate the effectiveness of computer vision in accurately assessing and quantifying the greening rate of green roof landscape plants in ocean cities. The intelligent interaction of computer vision in image processing has proven to be highly beneficial in evaluating and optimizing the integration of green roof landscape plants, leading to a more sustainable and aesthetically pleasing urban environment. The specific results of the analysis are as follows:

(1) Analysis of Plant Landscape and Human Interaction:

The rooftop garden of Alibaba’s headquarters building features a diverse plant landscape carefully curated to provide both aesthetic value and functional benefits. The designers employed 3D modeling and VR technology to create an immersive experience for users and stakeholders. By conducting in-depth case studies, the analysis revealed a strong alignment between computer vision and green rooftop landscape plant design in marine cities, further emphasizing the significance of intelligent interaction design and computer visual design techniques [33].

(2) The plant landscape design in the rooftop garden incorporates four functional modules: negotiation space, observation deck, multi-person party area, and VR virtual experience zone. Each module is strategically placed to enhance the interaction between visitors and plants, fostering a deeper connection with nature. The rooftop garden features a variety of plant species, including weeping willow, camphor trees, magnolia, cattails, and more, each chosen to create a specific ambiance and offer a range of sensory experiences.

The design also considers the rooftop garden’s accessibility, line-of-sight permeability, boundary richness, and interactivity. For instance, the semi-open waterfront space is adorned with water’s-edge plants like lotus and cattails, creating a beautiful and natural water landscape that allows for close interaction with the garden. Meanwhile, the semi-enclosed space is densely planted with yew, magnolia, and camphor trees, forming a hidden and secluded landscape, providing visitors with a sense of tranquility and enclosure.

(3) Plant Landscape Lighting Design and Human Interaction:

The rooftop garden’s plant landscape lighting design significantly contributes to the interactivity between people and the environment, particularly during nighttime hours. This lighting design takes into account human physiological and psychological characteristics, emphasizing a human-centric approach. By thoughtfully selecting light sources and lamp positions based on plant height and characteristics, the lighting design effectively showcases the rooftop garden’s unique features [34].

Using light superposition, flow, translucency, and refraction, the plant landscape lighting design creates a mesmerizing visual experience that captivates visitors. The lighting fixtures are strategically hidden through various methods, allowing the light to flow through the foliage or create enchanting silhouettes, fostering a harmonious connection between light and nature.

The lighting design also adapts to the changing seasons and weather conditions, allowing visitors to experience the rooftop garden’s different scenes throughout the year. During spring, the focus is on illuminating lush crowns and leaves, while winter highlights the striking branches and tree trunks. This thoughtful adjustment enhances the rooftop garden’s interactivity, making visitors feel closer to nature and creating a sense of wonder and awe.

In conclusion, the application of computer visual design techniques, 3D modeling, and VR technology in the intelligent interaction design of green roof landscape plants in ocean cities offers numerous benefits. From accurately assessing the greening rate to curating a diverse and interactive plant landscape and employing sophisticated lighting design, these intelligent technologies enhance the overall experience of green rooftop gardens. As urbanization continues, these findings provide valuable insights for designers, policymakers, and researchers seeking to create sustainable and engaging green spaces in ocean cities.

## 4. Comparative Analysis of Design Effects: A Case Study of Three-Dimensional Technology in Green Roof Landscapes of Coastal Cities 

### 4.1. Case Selection Criteria and Analytical Methods

In line with current design trends for green roof landscapes in coastal cities, the dominant approach is visual design using new 3D2020 software. Through analysis of the climate conditions in marine cities, we aimed to understand the optimal design of roof gardens, including spatial requirements and plant arrangements. We used interactive software to simulate visitors’ movements within the garden and generate ideas for an interactive experience. Abbreviations are defined on first use. In this paper, we examine the process of designing green roof landscapes using 3D software. We focus on two representative cases and conducted a comparative analysis with three additional cases to enhance the results. To gather information for the 3D design, we conducted a literature survey and used a specialized program design. According to the research cases presented in the previous section, this section analyzes the three-dimensional effects of various forms of green roofs. The following Table 2 contents are organized accordingly.

### 4.2. Landscape Plant Analysis after Applying 3D Technology

This papers analyze four representative green roofs for their three-dimensional effects and uses 3D and VR technology for further explanation. Table 3 shows the design of Alibaba’s roof garden landscape plants.

The rooftop garden landscape design for the 361° office building in Xiamen is as displayed in Table 4.

As indicated in Table 5, the landscape design of the rooftop garden in Qingdao Central Business District was created.

### 4.3. Summary of Analysis

As presented in Table 6, a comparative analysis was conducted to examine the impact of 3D technology in the three primary scenarios. The analysis summarizes the application space, advantages, and drawbacks for each case.

The three case studies of green roof development yield the following conclusions:

(1) It is necessary to enhance the theory of roof garden design by adhering to correct design.

First, the concept of appropriate ecological design is based on the climatic conditions of the region and selecting vegetation that suits the roof’s planting site. Second, the concept of sustainable development design is also important. Rooftop garden landscape design is a viable option, as shown by Alibaba’s success story. However, it is not necessary to adopt all popular foreign elements, which can increase costs and be an imprudent behavior. It is important to consider the impact on expenses before making such decisions. To ensure the seamless integration of such imported goods into China’s urban development, it is also essential to implement a sustainable development strategy for rooftop gardening. This must be carried out in a progressive and steady manner, taking into account national conditions and economic development.

(2) It is crucial to carefully select plants that are suitable for the region’s growth and development.

Firstly, the high location of the roof suggests the choice plants that thrive in direct sunlight and have a well-developed root system. Secondly, given the limited weight-bearing capacity, it is advisable to opt for lightweight flowers or herbaceous plants. Thirdly, since the soil layer on the roof is shallow and lacks water-storage capacity, drought-resistant vegetation is a suitable choice.

(3) Computer-aided three-dimensional technology can be utilized to optimize the landscape design in accordance with the building’s form and function.

The landscape design for the roof garden failed to consider the building group’s shape and did not establish clear design standards beforehand. To enhance the design program, it is recommended to lay out the landscape design in accordance with the building group’s shape and function. The intention of this approach is to adapt to the local conditions. Combined with the form and function of the building complex, a garden landscape must be designed that is harmonious with the surrounding environment and fully reflects the functional characteristics of the building. It must adopt a natural layout that reflects the coordination and integration of the building with the garden landscape or adopt a conventional approach that emphasizes landscape decoration and arrangement to enhance the landscape level and compensate for spatial constraints. However, in selecting garden vignettes, weight and volume restrictions due to the roof’s load-bearing capacity necessitate a considered approach. Alternatively, the designer may change the materials used for garden vignettes, such as employing lighter volcanic rock for rockeries. Adjusting the landscape of the roof garden can be achieved through manipulating color, shape, proportion, and texture by means of a computer.

### 4.4. Analysis of Results

(1) Generalization and General Design of Analytical Studies:

A fundamental consideration in research involving case studies represents the extent to which the findings can be extrapolated to wider contexts, thereby contributing to universal design principles and practices. In the context of the study titled “Computer Vision Interaction Design in Sustainable Urban Development: A Case Study of Roof Garden Landscape Plants in Marine Cities”, it is pertinent to address the generalizability of the case study findings.

The case study focused on the rooftop garden of the Alibaba headquarters building, examining the integration of computer visual design techniques, 3D modeling, and VR technology in the intelligent interaction design of green roof landscape plants. While this case study is insightful and provides a specific example of effective integration, it is essential to discuss the potential for broader applicability. Therefore, this study comparatively analyzes the roof gardens of two other ocean cities. The study of three cases makes the research conclusion more rigorous and scientific.

Universal design implications: The insights derived from the case study hold potential implications for the broader domain of green roof landscape design in ocean cities. The principles and methodologies employed, such as accurately assessing greening rates, curating diverse plant landscapes, and employing sophisticated lighting design, can serve as foundational concepts applicable to other similar projects. At the same time, comprehensive design is carried out from aspects such as color, shape, proportion, texture, and so on.

Key considerations for generalization: It is crucial to recognize that the success and generalizability of case study findings depend on various factors, including the context, scope, and specific characteristics of the target projects. The generalizability of our findings rests on the alignment of factors such as climate, geographical location, urban planning regulations, and user preferences.

While our case study offers a specific example of intelligent interaction design in a green roof landscape, its generalizability to universal design principles requires careful consideration of contextual factors. Our study serves as a foundation, and its findings can be interpreted alongside existing research to ascertain their broader applicability in enhancing green roof landscapes in various marine cities.

(2) Analysis of the Spatial Structure Presented in the Case Study:

Landscape typology of the rooftop garden: The landscape typology of the rooftop garden can be categorized as a “Multifunctional Urban Oasis”. This typology encapsulates the fusion of ecological, aesthetic, and interactive elements within an urban context. The garden serves as a retreat within the bustling city, offering not only visual delight but also functional benefits such as temperature regulation, air purification, and biodiversity enhancement. The negotiation spaces, observation deck, party area, and virtual experience zone each contribute to this multifaceted typology, providing opportunities for relaxation, connection with nature, and engagement.

The case study centers around the rooftop garden of the Alibaba headquarters building, a landmark in Hangzhou, China. This case is compared with the other two cases. This garden showcases a thoughtfully designed spatial structure that integrates various functional modules, plant species, and interactive elements. The spatial arrangement exhibits a purposeful division of zones, each contributing to the overall user experience and interaction with the green roof landscape.

Scope of application of the case study: The scope of this case study is centered around showcasing the integration of intelligent technologies, specifically computer visual design techniques, in the design and interaction of green roof landscapes in ocean cities. While the case study’s primary focus is on the rooftop garden of the Alibaba headquarters building, its implications extend beyond this specific location. The principles, methodologies, and findings presented offer valuable insights for designers, researchers, and policymakers seeking to enhance green roof landscapes in diverse marine cities facing similar urbanization and sustainability challenges.

By utilizing computer vision and 3D rendering techniques, the study addresses key aspects of green roof design: greening rate assessment, plant species composition, interactive design, and lighting considerations. The case study’s scope of application encompasses not only rooftop gardens in marine cities but also potentially any urban environment that aims to leverage intelligent technologies for sustainable and engaging green spaces.

## 5. Discussions

Prior this study, existing research has explored various aspects of green roof landscapes and their benefits in urban environments. Many studies have focused on the environmental advantages of green roofs, such as their ability to mitigate heat-island effects, improve air quality, and reduce stormwater runoff [35]. Additionally, some research has investigated the impact of green roof landscapes on the well-being and mental health of urban dwellers, highlighting the importance of incorporating nature into urban settings. Moreover, previous works have discussed the role of technology, including computer vision and 3DMAX modeling, in landscape design and urban planning. These technologies have been applied in different contexts, such as simulating urban environments, visualizing architectural designs, and assessing the performance of green spaces [36]. However, there is a gap in the literature regarding the specific application of computer visual design techniques, 3D modeling, and VR technology in the intelligent interaction design of green roof landscape plants in ocean cities. This study seeks to bridge that gap by exploring the novel use of these technologies in the context of rooftop gardens. This study stands out from the existing research in several key aspects [37]. Firstly, it addresses the specific context of green roof landscapes in ocean cities, which presents unique challenges and opportunities compared to other urban settings. Ocean cities face distinctive climate conditions, and their rooftop gardens must be carefully designed to withstand saltwater exposure and other environmental factors [38]. This paper acknowledges these challenges and proposes intelligent interaction design solutions that cater to the marine city context.

Secondly, this research places a strong emphasis on the integration of intelligent technologies, particularly computer visual design techniques, 3D modeling, and VR technology, in the design process. By leveraging these tools, designers can interact intelligently with the rooftop landscape, leading to more informed decisions and better outcomes. This integration of technology and design aligns with the growing trend of smart urban development and highlights the potential for creating innovative and sustainable green roof landscapes [39].

While this study provides valuable insights into the application of computer visual design techniques and intelligent interaction design in green roof landscapes, there are certain areas that warrant further exploration. Firstly, the research primarily focuses on the case study of the Alibaba headquarters building. Although there are also comparative analyses of two other cases, this may limit the generalizability of the findings. Future research could include a broader range of case studies from various ocean cities to enhance the comprehensiveness of the results.

Additionally, the study primarily emphasizes the greening rate and aesthetic aspects of rooftop gardens. Future research could delve deeper into the functional aspects of green roof landscapes, such as their role in providing habitat for biodiversity, supporting urban agriculture, and contributing to energy efficiency and building performance. Understanding the multifaceted benefits of rooftop gardens will enable a more holistic approach to their design and integration into urban environments. As technology continues to evolve, there may be opportunities to explore emerging computer visual design techniques and other intelligent technologies that could further enhance the interaction design of green roof landscapes. Integrating advancements in artificial intelligence, data analytics, and sensor technologies could open new avenues for optimizing the performance and user experience of rooftop gardens.

## 6. Conclusions

The conclusions drawn from this research are well defined within a focused and referenceable research scope. Through a comprehensive analysis of the rooftop garden at the Alibaba headquarters building and a comparative analysis of the other two cases, this study effectively demonstrates the applicability and benefits of integrating computer visual design techniques, particularly 3D modeling and virtual reality, in the intelligent interaction design of green roof landscape plants in marine cities. This article studied the intelligent interaction of green rooftop landscape plants in marine cities using computer vision technology. This can enable more reasonable design of landscape plant layout. This article tested the distribution of greening rates of various rooftop landscapes under computer vision through experiments. These greening rate data can provide a reference for landscape design. This research delves into the realm of intelligent interaction design of green roof landscape plants in ocean cities, leveraging the power of computer visual design techniques and 3D modeling. The rapid urbanization and the growing demand for sustainable development have given rise to the emergence of green roof landscapes, which not only provide significant environmental benefits but also contribute to the overall well-being of urban dwellers.

The integration of intelligent technologies is paramount in optimizing the design and interaction experience of green roof landscapes. Through case studies and extensive analysis, we have established that computer vision, particularly in the image processing of rooftop landscapes, offers remarkable accuracy in assessing and quantifying the greening rate of green roof landscape plants. This valuable information empowers designers and policymakers to create more effective and sustainable urban environments.

The application of 3DMAX modeling and VR technology enriches the design process by providing designers, users, and stakeholders with immersive and engaging experiences. By simulating and visualizing the rooftop gardens, designers can interact intelligently with the space and make well-informed decisions to enhance the landscape design effectively. The compatibility between computer vision and green rooftop landscape plant design in marine cities underscores the potential of these technologies in shaping aesthetically pleasing and functional green spaces.

The specific conclusions obtained within this scope include the following:

Effectiveness of computer vision design aids: The efficiency of computer vision design technology is prominently demonstrated through its utilization in the analysis of greening rates via 3DMAX renderings. This approach offers a precise and dependable method for assessing and quantifying the extent of greenery within rooftop landscape plants. Moreover, within the domain of plant landscape design, the versatility of this technology allows for real-time adjustments to design schemes in alignment with specific requirements. As a result, the overall efficacy of the design process is significantly enhanced.

Alignment with design: The alignment between computer visual design techniques and green rooftop landscape plant design in marine cities is clearly demonstrated. Through in-depth case studies, we highlight the significance of intelligent interaction design and computer visual design techniques in enhancing user experiences with green rooftop gardens.

Scope of application: Our study’s conclusions are specifically applicable to the integration of computer visual design techniques, 3D modeling, and VR technology in green roof landscaping within ocean cities. The findings provide valuable insights for designers, policymakers, and researchers working on sustainable urban development in similar marine environments.

Enhanced interaction design: Through in-depth case studies, the study affirms a strong alignment between computer visual design techniques and the effective design of green rooftop landscapes in marine cities. The utilization of 3D modeling and virtual reality facilitates immersive experiences for designers, users, and stakeholders. This finding underscores the potential for intelligent technologies to enhance human interaction with green spaces.

Ecological considerations: The research recognizes the importance of plant species composition and ecological balance in green roof landscape design. The study’s application of computer vision in identifying and categorizing plant species informs the selection process for plants that thrive in marine climates, thus contributing to sustainable and resilient designs.

User-centered approach: The investigation underscores the significance of a human-centric approach in landscape lighting design. By thoughtfully adapting lighting to seasonal changes and physiological factors, the research showcases the potential of computer visual design techniques to create captivating visual experiences that foster a stronger connection between visitors and the natural environment.

Applicability to ocean cities: The study’s scope is clearly defined within the context of marine cities, catering to the specific challenges and opportunities posed.

## Figures and Tables

**Table 1:** Proportion of park landscape color carriers.

Type	Name	Description	Benefits	Pictures
Shrubs	Dwarf Pomegranate (*Punica granatum* ‘Nana’):	Dwarf pomegranates are small shrubs with glossy leaves and vibrant orange-red flowers that resemble miniature pomegranate blossoms.	These shrubs add a burst of color to rooftop gardens, and some varieties even produce small edible fruits. They thrive in full sun and can be pruned to maintain a compact size suitable for containers.	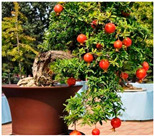
	Lavender (*Lavandula* spp.)	Lavender is a fragrant herb with slender leaves and spikes of purple or blue flowers.	Lavender adds a delightful aroma and attracts pollinators like bees and butterflies. It thrives in sunny, well-drained locations and can be used for culinary purposes or for crafting aromatic products.	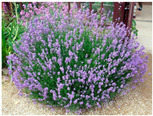
	Rosemary (*Rosmarinus officinalis*)	Rosemary is an aromatic herb with needle-like leaves and small blue flowers.	Rosemary is drought-tolerant and well-suited for rooftop gardens. It adds fragrance and culinary versatility, and its evergreen foliage remains attractive year-round.	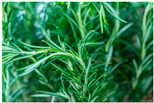
Ground Covers	Blue Oat Grass (*Helictotrichon sempervirens*):	Blue oat grass is an ornamental grass with striking blue-gray foliage that forms neat clumps.	This grass offers a unique color contrast in rooftop gardens. Its compact size and drought tolerance make it suitable for containers. The foliage sways gracefully in the wind, adding movement to the landscape.	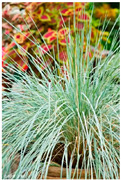
	Creeping Jenny (*Lysimachia nummularia*)	Creeping Jenny is a trailing perennial with round, bright yellow-green leaves.	Creeping Jenny forms a lush ground cover that spills over edges, softening the look of containers. It is excellent for preventing erosion and adds a pop of color to your rooftop garden.	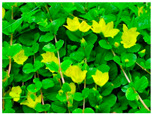
	Japanese Forest Grass (*Hakonechloa macra*)	Japanese forest grass is a low-growing ornamental grass with cascading, arching foliage.	This grass adds a graceful and flowing element to rooftop gardens. Its shade tolerance makes it suitable for partially shaded areas, and its texture creates a soothing contrast with other plant types.	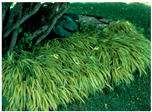
Trees	Japanese Maple (*Acer palmatum*)	Japanese maples are small trees known for their delicate, finely divided leaves and stunning fall colors.	Japanese maples bring elegance and architectural interest to rooftop gardens. They are available in various sizes and leaf colors, allowing for customization. Their foliage provides shade and their vibrant hues add seasonal beauty.	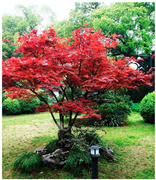
	Dwarf Olive Tree (*Olea europaea* ‘Little Ollie’)	Dwarf olive trees are small, evergreen trees with silvery leaves and a classic Mediterranean appearance.	These trees bring a touch of the Mediterranean to rooftop gardens. They can be grown in containers and provide a timeless and sophisticated aesthetic. The silvery leaves reflect sunlight, helping to reduce heat absorption.	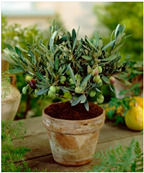
	Dwarf Weeping Cherry (*Prunus subhirtella* ‘Pendula’)	Dwarf weeping cherry is a compact tree with cascading branches and delicate pink or white blossoms.	This tree brings a touch of elegance and romance to rooftop gardens with its pendulous branches and beautiful spring flowers. Its smaller size is suitable for containers, and it can be pruned to maintain its shape.	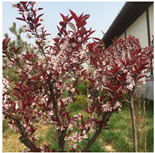

**Table 2:** Case information.

No	Device Name	Located in a City
A	Alibaba Headquarters Landscape Design	Shanghai, China
B	Roof Garden Landscape Program Of Xiamen 361 Office Building	Xiamen, China
C	Roof Garden of Qingdao Central Business District	Qingdao, China

**Table 3:** Alibaba Headquarters Landscape Design.

Overview		
Setting up a green space that is distinctively characterized by a landscape corridor, theater area, and leisure space in the rooftop garden can provide the city landscape with a living-room function. Various plantings can be combined and echoed to provide green spaces that promote the sustainable development of the ocean city.		
Photograph	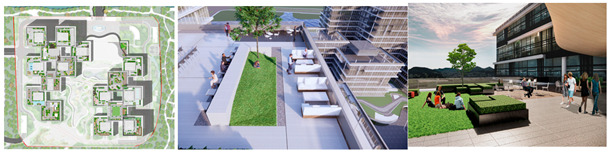	
Application characteristics of 3D technology		
Color	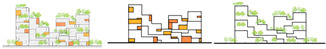	The green roof space was functionally divided with various colors representing different functions paired with different types of plants.
Shape	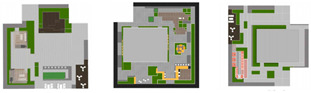	According to the various sizes and orientations of green roof spaces, the morphological design satisfies both mobility and ornamental needs, utilizing plant shading to divide the area.
Proportion	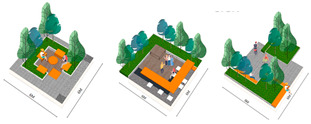	The green roof’s planting ratios were determined by the space proportions of the site, accompanied by corresponding decorative proportions for each area.
Texture	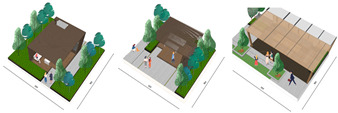	Through the use of plant texture matching, ground pavement, installation styles, wall decorations, and other textures, the embodiment of different effects of three-dimensional technology was achieved.

**Table 4:** Roof Garden Landscape Program of Xiamen 361° Office Building.

Overview		
The 361° Building is situated in Xiamen, China, a typical seaside city. In general, it employs a new Chinese style of landscaping, incorporating various types of flora based on population usage, emphasizing a more upscale and tasteful ambiance, and showcasing Zen influences.		
Photograph	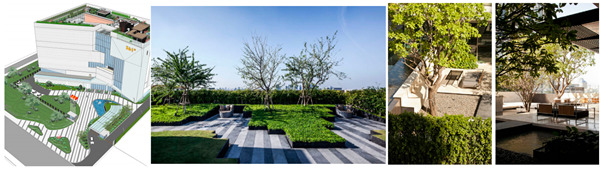	
Application characteristics of 3D technology		
Color	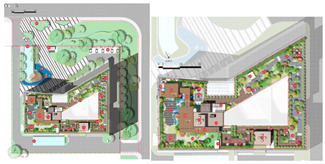	Computer software was utilized to differentiate the colors on the roof plan according to their respective functions. The vegetation colors can also contribute to the division of space as well as the paving materials.
Shape	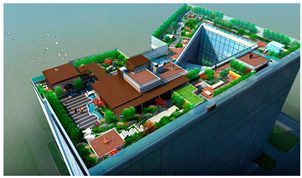	Based on the building’s brand image, a stylized plant design was implemented in the rooftop garden. The planting space was designed to reflect the brand’s colors and shapes.
Proportion	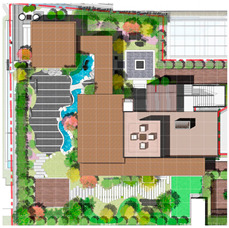	The design of the plants and the spatial layout of the rooftop garden must adhere to ergonomic principles. The size of the plants and the spatial scale should be proportional to achieve a harmonized design.
Texture	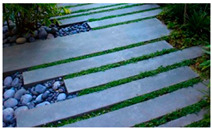	In the hardscaping section of the rooftop garden, it is important to maintain an overall unity in texture style. Combining hard slate and soft plants creates an intriguing and beautiful textural contrast.

**Table 5:** Roof Garden of Qingdao Central Business District.

Overview		
The rooftop garden design at Qingdao Central Business District is based on the concept of small scale. The combination of compact space and foliage creates an ideal walking and leisure area.		
Photograph	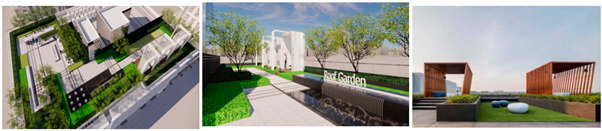	
Application characteristics of 3D technology		
Color	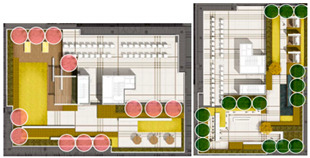	The rooftop garden design in Qingdao’s Central Business District is based on the concept of a smaller scale, featuring proportionate space and plants that together create a rooftop garden suitable for walking and leisure.
Shape	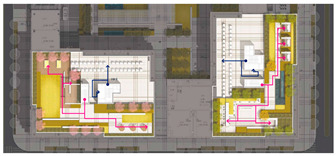	The design of the plant layout for the roof garden space is based on the image of sparse and dense urban transportation networks, resulting in a rhythmic plant network with an irregular shape.
Proportion	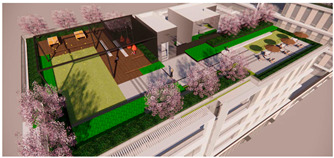	The design of green roof plants fully takes into account the size ratio of plants, utilizing it to create varying smaller spaces. No changes necessary.
Texture	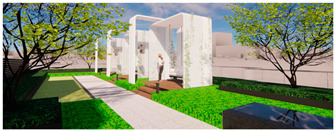	The floor paving texture in the rooftop garden complements the texture of the plants, contributing to the harmonious spatial design.

**Table 6:** Comparative analysis of rooftop garden 3D technology application cases.

	Case	Application Space Type	Advantage	Insufficient
A	Alibaba Headquarters Landscape Design	Office building roof garden	Public space; large site scale; rich plant mix.	The ornamental value is average; the plant space is fragmented.
B	Roof Garden Landscape Program Of Xiamen 361° Office Building	Roof garden of sports brand building	Chinese cultural elements have a strong sense of experience; there are many types of plants, and the design effects are rich.	The dredging and discreteness are weak; large green plants cannot be planted.
C	Roof Garden of Qingdao Central Business District	Rooftop garden in business district	Strong sense of ecological experience; strong sense of plant design.	The ornamental value is single; the planting of some plants in the northern region is limited.

## Data Availability

Not applicable.
